# Hearing Screening for Congenital CytoMegaloVirus—Exploring Parents’ Experiences of Completing Targeted Congenital Cytomegalovirus Screening at the Time of Their Infants’ Newborn Hearing Screening

**DOI:** 10.3390/jcm13154367

**Published:** 2024-07-26

**Authors:** Emma Webb, Jan Hodgson, Alanna N. Gillespie, Cheryl A. Jones, Zeffie Poulakis, Janis Wong, Valerie Sung

**Affiliations:** 1 Murdoch Children’s Research Institute, Melbourne, VIC 3052, Australia; emma.webb@mcri.edu.au (E.W.);; 2 Department of Paediatrics, The University of Melbourne, Melbourne, VIC 3052, Australia; 3 Medicine and Health, The University of New South Wales, Sydney, NSW 2050, Australia; 4 Sydney Children’s Hospital Network (Westmead), Sydney, NSW 2145, Australia; 5 The Royal Children’s Hospital, Melbourne, VIC 3052, Australia; 6 Faculty of Medicine, The University of Melbourne, Melbourne, VIC 3052, Australia

**Keywords:** congenital cytomegalovirus, universal newborn hearing screening, hearing loss, targeted screening, qualitative research

## Abstract

**Background/Objectives:** Congenital cytomegalovirus (cCMV) is the leading infectious cause of sensorineural hearing loss and neurodevelopmental disabilities, with prompt detection (<21 days of life) required to enable accurate diagnosis and anti-viral treatment where clinically appropriate. International guidelines recommend cCMV screening for infants who do not pass their Universal Newborn Hearing Screening (UNHS). This study aimed to explore parental experiences of targeted cCMV screening through the UNHS in Victoria, Australia between 2019 and 2020 (HearS-cCMV study). **Methods**: A qualitative study comprising 18 semi-structured interviews with parents who took saliva swabs from their infants who did not pass their UNHS. A maximum variation sampling strategy was used with data analysed using thematic analysis. **Results**: Four themes described 18 parents’ experiences of cCMV screening: (1) parents’ lack of CMV awareness prior to cCMV screening; (2) overall positive experience; (3) varied understanding of CMV post screening; and (4) parents were glad to screen their infant for cCMV. Enablers of targeted cCMV screening included the swab being simple and non-invasive, being easier to complete in the hospital than at home, and the screening being well delivered by the staff. Barriers included a potential increase in anxiety, especially with false positives, and the timing of cCMV screening coinciding with their infant not passing UNHS being difficult for some parents. **Conclusions**: Parent experiences of targeted cCMV screening were positive. Increasing public knowledge of cCMV and training staff members to complete the CMV swab would reduce the risk of false positives and associated parental anxiety. This would facilitate successful routine targeted cCMV screening.

## 1. Introduction

Congenital cytomegalovirus (cCMV) is the leading infectious cause of sensorineural hearing loss and neurodevelopmental disabilities [1,2]. As well as hearing loss, cCMV can be the cause of other newborn concerns such as growth retardation, prematurity, and neurological abnormalities as well as long-term impacts such as vision loss, intellectual impairment, and cerebral palsy [3,4]. Oral anti-viral therapy, valganciclovir, if commenced within the first month of life, has been shown to stabilise hearing and improve neurodevelopmental outcomes in symptomatic infants [5] and is recommended for infants with symptomatic cCMV at birth [2]. Recent guidelines have advised for antiviral treatment to be an option for infants with cCMV-related isolated SNHL [6]. The preferred method of diagnosing cCMV using salivary CMV polymerase chain reaction (PCR) testing must be completed within 21 days of life [7] to differentiate from post-natally acquired CMV infection, which is generally not known to cause hearing loss or neurodevelopmental disabilities [8], with some suggestion of adverse outcomes in extremely premature infants (<32 weeks) such as sepsis [9].

Guidelines around the world recommend targeted cCMV screening within 21 days of life for newborns who do not pass their Universal Newborn Hearing Screening (UNHS) [2,6,10,11,12]. The UNHS aims to detect congenital hearing loss in neonates and is completed in many developed countries [2]. Targeted screening of cCMV at the time of the UNHS aims to identify infants with cCMV-related SNHL who would otherwise miss the 21-day timeframe for an accurate diagnosis through the use of urine or saliva PCR analysis, and consequently, potential anti-viral treatment for appropriate infants [6]). Of the 85–90% of infants who are asymptomatic at birth [1,13], approximately 10–20% will go on to have delayed-onset SNHL [10,14]}. These infants would not be identified through targeted cCMV screening at the time of the UNHS. Given that most asymptomatic infants do not develop any long-term symptoms, this raises the ethical question of whether universal screening (all infants screened) for cCMV should be performed in the absence of appropriate treatment for these infants [6], and the potential burden of parental anxiety in this situation [5].

A targeted cCMV screening program at the time of the UNHS allows for accurate diagnosis within 21 days of life and appropriate infants who have confirmed SNHL to be considered for timely anti-viral treatment within the first month of life [5]. Despite international guidelines recommending targeted cCMV screening for infants who do not pass the UNHS, practices for cCMV screening vary around the world. Neither universal screening nor targeted screening for cCMV at the time of the UNHS is completed routinely, with some centres around the world offering one or the other. One study in Australia found it feasible to deliver targeted cCMV screening at the time of the UNHS [15]. Two studies in Australia and the United Kingdom have found it both feasible and acceptable from a parental perspective to complete targeted cCMV screening at the time an infant does not pass their UNHS, using quantitative research such as short surveys to determine acceptability [16,17]. This was found to be the case in a clinical setting where the swab was performed at the hospital by either the newborn hearing screeners or parents [16] or by parents only in a hospital setting or at home [17]. A study in the US in 2011 [18] assessed parental attitudes towards hypothetical cCMV screening and demonstrated that for most parents, costs, worry, and anxiety associated with newborn screening for CMV would be acceptable if targeted cCMV screening was implemented, but there was a minority of parents who opposed the idea of screening for cCMV. A more recent study [19] completed three years following the implementation of routine targeted cCMV screening in Utah, US, aimed to explore caregivers’ knowledge and attitudes about cCMV screening through a survey completed by caregivers of children waiting in a paediatric ear, nose, and throat specialist’s waiting room. This study found a generally positive attitude towards screening for the virus with most caregivers supporting cCMV screening; however, the reasons underpinning parent support were not explored.

The decision on whether to implement newborn screening for cCMV infection should be based not only on the expected benefits but also on the expected costs and harms [18]. To ensure such screening programs reflect the realities of community and practice settings, and to determine how acceptable it is to these members of the community, it is essential that practitioners and community members be involved in conceptualising and designing feasibility research [20]. The newborn period can often be an overwhelming and busy time for parents, and there are many health conditions that can be screened for in the newborn period in addition to hearing, including heart defects and biochemical genetic disorders [21]. In 1968, Wilson and Jungner [22] developed the “Principles and Practice of Screening for Disease”, highlighting 10 criteria that should be considered when implementing screening practices. In 2020, Haller [23] examined targeted cCMV screening in line with the 10 criteria from Wilson and Junger [22] and stated there is evidence and rationale supporting targeted cCMV screening at the time of the UNHS with suggestions for future research highlighted. Criterion 5 highlights that the “test should be acceptable to the population”. As outlined in Haller’s work [23], an assessment of parental acceptability of screening for cCMV showed that the population was generally in favour of testing for cCMV [19] but little was understood as to why.

Qualitative methods are valuable and utilised in implementation science to enable researchers to understand and address the barriers and facilitators to implementing evidence-based practice [24]. To our knowledge, no one has explored parental experiences and the barriers and enablers of targeted cCMV screening. This will provide vital information about the best strategies for facilitating the targeted cCMV screening programs that are now recommended. Our study aimed to explore parental experiences of targeted cCMV screening, within the Victorian UNHS context, to help understand factors facilitating and preventing early targeted cCMV screening. This program was delivered through the Victorian UNHS, the Victorian Infant Hearing Screening Program. Our research question was “What are the parents’ experiences of participating in a targeted cCMV screening program at the time their infant does not pass their newborn hearing screen?”

## 2. Materials and Methods

Based on the research question, we undertook a multi-centre qualitative study between February and August 2020. We used a phenomenological approach to explore the individual parental experiences and perceptions [25]. The study was approved by the RCH Human Research Ethics Committee (HREC/18/RCHM/273) and is reported according to COREQ.

### 2.1. Sampling

To capture a broad range of parental experiences, we used purposive maximum variation sampling [26] that aims to vary the characteristics of the participants. These characteristics include age, the sex of the parents, the hearing status of the infant, the hospital where the infant underwent the UNHS, and the location of the CMV swab completion and cCMV screening status.

### 2.2. Participants

Participants were recruited from four maternity hospitals in Melbourne, Victoria, Australia as part of the Hearing Screening for congenital CytoMegaloVirus (HearS-cCMV) screening program [17]. Parents of infants who did not pass the UNHS, which in Victoria, Australia, is a two-staged automated auditory brainstem response screening protocol, and were referred to diagnostic audiology, were invited to participate in additional salivary cCMV screening. Further details of the HearS-cCMV study are provided elsewhere [17]. Parents who returned the salivary cCMV swab for analysis and completed and returned the Participant Information and Consent Form were invited to participate in a short open-ended survey asking their thoughts on the cCMV screening program. These parents were also invited to express their interest in participating in a 30-to-60-minute semi-structured interview either via telephone or Zoom.

### 2.3. Data Collection

An interview guide was developed based on previous readings and clinical knowledge to help structure the semi-guided interviews (Appendix A). The script was piloted and revised by a staff colleague who was a parent and not involved in the study. During interviews, parents were encouraged to elaborate and discuss elements that were important to them. Live captioning was available during the interviews, completed by an external captioning company, as approved by the RCH Human Research Ethics Committee. Parents were made aware of this prior to the interviews at the time of the initial phone call to organise the interview, and again at the commencement of the interviews.

### 2.4. Data Analysis

The interviews were audio-recorded, transcribed verbatim, and validated by two members of the research team who de-identified participants and checked the transcripts against the original recording for accuracy. NVivo V.12 computer software was used to manage the data. The interview transcripts were analysed using the six stages of thematic analysis as detailed by Braun and Clarke [27]. EW completed the initial six steps of familiarisation with the data, identification of the initial codes, and searching for the themes. Reviewing the themes, defining and naming the themes, and completing the final analysis were completed in consultation with JH and VS as experienced qualitative, evidenced-based researchers. The research team had regular meetings to review and discuss the emerging themes until saturation of the themes occurred [28].

## 3. Results

**Figure 1:**
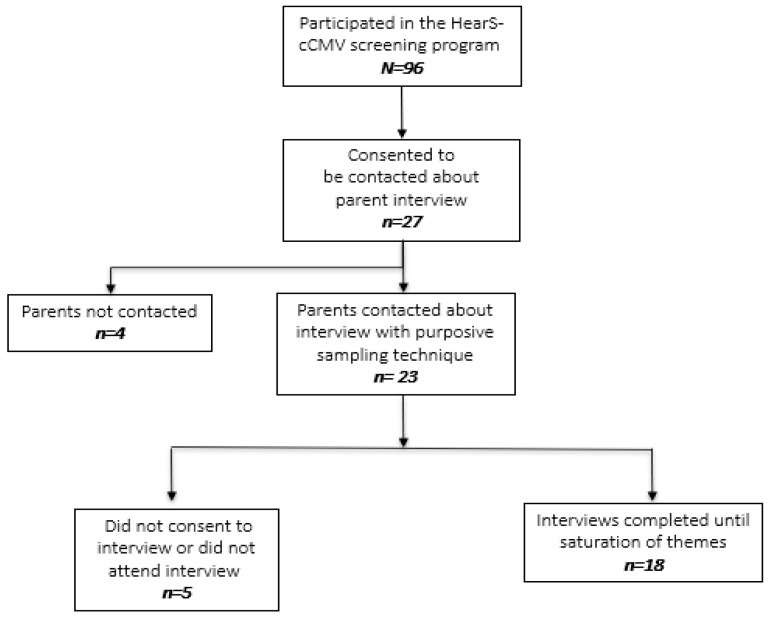
Sampling for parent interviews.

Of the 96 families involved in the HearS-cCMV screening program, 27 parents expressed interest in being contacted about participating in the interviews, and 18 parents participated in the semi-structured interviews (Figure 1). Demographic information is shown in Table 1. Of the 18 parents who participated in the interviews, 10 (55.5%) reported that their infant had abnormal hearing following their diagnostic audiology appointment (Table 1). Of the 79 parents who did not participate in the interviews, 22 (27.8%) had infants with abnormal hearing following their diagnostic audiology appointment. One infant with hearing status unknown had both parents participate in the interviews. Most parents who participated in the interviews had infants who screened negative for cCMV; one had a true positive cCMV diagnosis and two had a false positive cCMV diagnosis (Table 1). All parents whose infants received an initial positive CMV screening participated in the interviews. There were no asymptomatic cCMV infants in our study cohort, and therefore, we did not capture this cohort of families in the interviews. Interviews ranged between 30 and 60 min.

### 3.1. Parents’ Perceptions of the Newborn Period and Hearing Screening

Parents alluded to the newborn period being a very busy time, and they were offered a variety of different medical tests for their infant in the first few days of life. Overall, this period in the first few days of their newborn’s life was found by all to be somewhat overwhelming and emotional in nature.

*Just throw it in with all the other tests he had. ‘Cause he had all these blood tests done. All these everything done within the first four hours of life. You know, you—you squeeze his little foot and make him bleed.*

*(P15)*

Some parents reflected on what it felt like for them for their infant not to pass the hearing screen, which then resulted in the families being offered the additional cCMV screening.

*That information is hard for-- for parents. But there’s nothing wrong with the doctors who do the hearing screening because basically in general, it’s just a difficult message that parents were not prepared for.*

*(P10)*

### 3.2. Themes Derived from the Perspectives of Parents around the Targeted cCMV Screening

There were four key themes identified from the interviews: parents lacked awareness around CMV prior to cCMV screening, parents had an overall positive experience completing the cCMV screening, parental understanding of CMV post cCMV screening varied, and parents were glad they were able to screen their infant for cCMV. A detailed summary of the quotes for each theme is outlined in Appendix B. In addition, parents identified barriers and enablers to the cCMV screening program.

#### 3.2.1. Theme: Parents Lacked Awareness of CMV Prior to cCMV Screening 

Five parents specified that they had heard about CMV prior to the delivery of the cCMV screening program, due to working in the health industry or having medical concerns with their older children requiring investigation into potential cCMV. One parent mentioned that their GP provided them with information about CMV pre-pregnancy, and one parent indicated that they changed their behaviours while pregnant to avoid contracting CMV due to their knowledge of the virus and how it spreads. Some parents found that this lack of knowledge of CMV prior to the screening program made hearing about the cCMV screening program difficult for them.

*I think it was the-- not knowing what it (CMV) was, and then obviously the delivery of getting the information, because we didn’t know what CMV was at the time.*

*(P11)*

#### 3.2.2. Theme: Completing the cCMV Screening Was Overall a Positive Experience 

Parents found that generally the cCMV screening program was well delivered by all of the team involved and nearly all parents found the screening program easy and straightforward to participate in. The support and care from the UNHS staff were helpful, especially due to the staff’s understanding of the many challenges of the newborn period.

*Well, I think it was delivered really quite well, and it was actually very easy, very straightforward.*

*(P12)*

At times, having their infant not pass the UNHS made parents feel anxious or worried about their infant’s hearing, but they found the support from UNHS staff to be helpful in reducing this.

*I remember after completing the screening I got a follow up phone call and it was really really helpful in sort of helping with anxiety I had around her hearing loss.*

*(P7)*

Parents stated that the written and verbal information provided was clear, succinct, and helpful in understanding the cCMV screening process. Another one of the main contributors to parents’ positive experience of the cCMV screening program was that parents found the swab testing for CMV non-invasive, easy, quick, and straightforward.

*It wasn’t invasive at all. Of all the tests that (baby) had and still has, it was, yeah, easy.*

*(P13)*

At times, some parents felt that the benefits of the additional cCMV screening at the time of the hearing screening were not clearly highlighted, and as a result, they were unsure of how serious CMV was. Some parents discussed how the additional cCMV screening added to their anxiety levels and feeling overwhelmed about potential hearing loss and cCMV-related health concerns for their infant.

*So, the fact that it comes straight after a second fail. So, it’s not that the test itself is a real scary, I guess, but you’re already feeling overwhelmed because your child has just failed a second hearing test.*

*(P15)*

This was more so for parents who were told their infant had initially screened positive for cCMV and had to have repeat testing to confirm a true cCMV diagnosis. Despite some increased anxiety, parents were still grateful for the opportunity to test their infant for cCMV.

*I would say it was only kind of a small emotion of a bit of concern, vastly overridden by kind of gratefulness I suppose*

*(P5)*

#### 3.2.3. Theme: Parents’ Understanding of CMV Post CMV Screening Varied

Following participation in the cCMV screening program, understanding of the virus was varied between parents. Nearly half of the parents understood that CMV was a virus that could be passed to the fetus in utero, whereas a few understood that cCMV could cause hearing loss. Few parents recognised that they had limited knowledge and understanding of CMV following the screening.

*I cannot honestly say I know too much about it really or learned too much post-test I suppose. There was lots of other stuff to do at the time.*

*(P5)*

Parents who had limited understanding of CMV stated that this was due to their infants screening negative for CMV or having a normal hearing diagnosis after undergoing formal diagnostic audiology testing. There were variations in parents’ understanding of the cCMV screening timeframes, with some misunderstanding that screening needed to be completed within 21 days of age or that administration of anti-viral treatment if eligible needed to commence within one month of age.

#### 3.2.4. Theme: Parents Were Glad They Were Able to Screen Their Baby for cCMV

Parents appreciated that they were provided the opportunity to screen their infant for cCMV, with feelings of being grateful and reassured by the testing. Parents explained that this was due to being able to determine whether cCMV was or was not the cause of their infant’s hearing loss as well as providing other information about their infant’s health, such as ruling cCMV out of the cause of other undiagnosed health concerns.

*It was helpful that doing the test rules out one of the causes of hearing loss and about getting more information about why there is a hearing loss.*

*(P1)*

In relation to diagnosing cCMV, the parent receiving a true positive cCMV diagnosis felt that it was meaningful, as early screening allowed for treatment intervention, more medical support, and early intervention.

*It’s more having at least an indication of things to look out for. And because he had CMV, and all of the people that are giving him intervention, support, and caregiving know that he’s had CMV, they’re probably more vigilant about looking at other things other than the hearing loss.*

*(P11)*

When asked what parents’ thoughts were if they knew there was a test for cCMV and were not offered it, some parents stated that they would be disappointed or upset if they were not offered the cCMV screening. This was especially due to the potential treatment options available to help their infants’ hearing or other health issues if there was a confirmed diagnosis of cCMV.

*I think that I would be pretty angry. Especially if it is something that can cure the deafness. You would feel responsible that you did not know about it. So I think that I would be very angry. And very upset.*

*(P6)*

### 3.3. Barriers and Enablers to the cCMV Screening Program

Participants also identified barriers and enablers to the cCMV screening program with supporting quotes detailed in Appendix C and Appendix D. Enablers included that the cCMV swab was simple and non-invasive to complete, support from the staff was helpful, a high level of faith in the hospital staff, and the swab was easier to complete if performed in the hospital rather than at home. However, completing the cCMV screening was difficult at times for some parents coinciding with the timing of their infant not passing UNHS, and false positives could lead to an initial increase in anxiety.

## 4. Discussion

This is the first qualitative study to explore parental experiences of participating in a parent-completed targeted screening for cCMV at the time of their infant’s UNHS. Parents found this experience generally positive, and they were glad to be able to determine whether their infant had cCMV. For most parents, the cCMV screening was the first time they had heard about the virus. The lack of awareness of cCMV amongst parents who participated in the screening program is consistent with other research where much of the public is unaware of the virus [29,30] despite it affecting around 86% of women of reproductive age in the general population, with a higher CMV prevalence in developing countries compared to developed countries [31,32]. Unsurprisingly, parents indicated that they were already experiencing varying levels of anxiety and uncertainty due to their infant not passing their UNHS. The additional cCMV screening that was then offered led to some increased anxiety and feelings of uncertainty about the importance of whether to screen their infant for cCMV, with parental lack of knowledge of the virus being a strong contributing factor towards the decision around screening their infant for cCMV. This is consistent with findings from a survey completed in Utah which found that parents would be more in favor of cCMV screening if they had a greater level of knowledge of cCMV [19]. It is important to note that the families who did receive a positive screen result stated they felt much more informed about cCMV through this process, which can be attributed to the information provided by the medical team involved.

Whilst targeted cCMV screening at the time of the UNHS in Australia, the UK, and the US [33] has been deemed feasible and acceptable, regardless of whether the CMV swab was completed by hearing screeners [15,34] or by parents [17], there is little understood as to why the screening program is feasible and acceptable for parents. Themes derived from our interviews with parents provide some explanations, with parents having had an overall positive experience participating in the cCMV screening and finding the test simple and non-invasive. One enabler of screening was the support and knowledge of all staff involved, from the initial delivery of the screening program from the hearing screeners at the time the infant did not pass their UNHS, through to the infectious disease team at the time of a true cCMV diagnosis. A second enabler of screening was the swab being simple, straightforward, and non-invasive. Findings from a systematic narrative review, which assessed the acceptability of childhood screening programs, identified two main constructs for acceptability: firstly, parental understanding of the program, and secondly, how the parent feels about the program [21]. Carlton [21] stated that acceptability of screening increases when the amount of effort required for the parent to complete the intervention is minimal, such as location (whether it could be completed in the hospital or at home), and the discomfort of testing on the child.

For some parents, it was evident that the reassurance that their infant did not have this common virus despite no health concerns in the newborn period was helpful. Furthermore, parents were not only glad to determine whether cCMV was the cause of their infant’s hearing loss but also other unexplained health issues that their infant may have had. In the absence of clinically apparent symptoms at birth (85–90% of children with cCMV are asymptomatic at birth [1]) and the absence of routine targeted or universal cCMV screening, the main method of detection of cCMV relies on a retrospective analysis of newborn dried blood specimens through PCR testing [7]. However, limitations arise with this method given that it has a low sensitivity rate [7] and the fact that, often, the diagnosis will not occur within the required one month of life to allow for timely anti-viral treatment. This poses the risk of diagnostic odyssey for many families, a term relating to “protracted journeys towards diagnosis for people living with rare diseases” [35]. Given these aforementioned factors, it is not surprising that parents found it reassuring to know whether their infant had cCMV in determining whether this was the cause of their infant’s hearing loss or other health-related concerns and reducing the potential risk of diagnostic odyssey.

It is important to reflect on the benefits for families, which include ruling out cCMV as a cause of hearing loss or health issues, or a true cCMV diagnosis to allow for a diagnosis and timely treatment. However, the impacts of a false positive screen result must be considered. A false positive result in which a disease is predicted to be present when it is not can be an emotional experience for those involved [36]. As outlined in our feasibility and acceptability results for the targeted cCMV screening completed in Victoria [17], three false positive swabs were attributed to breast milk contamination and parental misunderstanding of when to complete the swab (at least one hour post breastfeeding) [7]. Parents who received the false positive screen result and completed the interviews highlighted that whilst this false positive resulted in an initial concern about their infant’s health and increased anxiety, the timely turnaround of confirmation of a false positive was highly reassuring, and they were still glad to have their infant screened for cCMV. This timely confirmation is an important component of the implementation of a successful screening program. One consideration is to ensure parents are educated at the time of the cCMV screening of the potential risk of false positives and the processes completed to confirm the cCMV status of the infant. Alternatively, staff members could be involved in completing the CMV swabs as opposed to parents as a method of reducing the potential of false positives from breastmilk contamination.

Enablers and barriers are important factors to consider when assessing the acceptability of screening programs. The enablers of the cCMV screening program mostly highlight the simplicity of participating in the program and completing the CMV swab with support from the staff. This was remarkable, considering the swabs were completed by parents rather than health care professionals, and parents had to post the swabs back if they were completed at home. Whilst most parents alluded to this being a simple process, many thought it would be simpler if this was completed in the hospital at the time of the second refer result on the UNHS and completed by staff members. This would overcome the barriers that include parental uncertainty of whether they have completed the swab correctly and ensure the correct timing of taking the swab if the baby is breastfed, to reduce the risk of false positives from breastmilk contamination. Completing the swab in the hospital would also reduce the need to post the swab back to the hospital when the swab is completed at home.

We have added to the literature by using a qualitative methodology and including a wide range of perspectives to enable a rich understanding of parental experiences participating in targeted cCMV screening. This included conditions of varying cCMV screening status, as well as including parents from all the participating hospitals, parents completing the swabs at home and in the hospital, parents of infants who had normal hearing and hearing loss, and parents of both male and female sex. We also included parents who had English as a second language and required an interpreter. Our study was limited to include only the perspectives of parents who participated in the screening. Although only a small proportion of families did not participate in screening (30/126, 24%), future research should also capture the perspectives of families who chose not to undergo a cCMV screening. An exploration of a cCMV screening program from the perspective of families whose infants are born with asymptomatic cCMV is also very important, from both the context of universal and targeted screening. However, this was unable to be captured in our study given there were no asymptomatic cCMV infants identified. A further study is underway to explore staff perspectives of the targeted cCMV screening program.

Our study is the first study to explore in-depth parental experiences of a targeted cCMV screening program at the time of the UNHS. It demonstrates the need to continue to improve public knowledge of CMV, as well as for improved knowledge of cCMV for parents at the time of participating in the targeted cCMV screening program. It also raises the consideration of training staff members to complete the CMV swab. This would not only reduce the risk of false positives from breastmilk contamination and associated anxiety but ensure swabs can be performed in the hospital and in turn reduce the burden for parents to complete this at home.

## 5. Conclusions

Our study has provided an in-depth and rich understanding of parental experiences of targeted cCMV screening at the time their infant does not pass their UNHS. Overall, cCMV screening was a positive experience for parents and they were glad to know the cCMV status of their infant. Consistent with the previous literature, a lack of cCMV knowledge prior to screening may lead to increased parental anxiety at the time of cCMV screening. Some parents also felt a lack of certainty about the importance of the screening program. The parental experiences reported here will inform strategies for the successful delivery of targeted cCMV in the Victorian context. It provides vital parent input to ensure that the future implementation of routine targeted cCMV screening meets the needs of parent stakeholders.

## Figures and Tables

**Table 1:** Parent characteristics.

Participant	Sex of Parent	Parents’ Understanding of Hearing Status of Infant at Time of Diagnostic Audiology	CMV Swab Location	cCMV Screen Result
1	Female	Profound SNHL	In-patient	Negative
2	Female	Mild SNHL	In-patient	Negative
3	Male	Profound SNHL	In-patient	Negative
4	Female	Moderate SNHL	Out-patient	Negative
5	Male	Normal hearing	In-patient	Negative
6	Female	Normal hearing	In-patient	Negative
7	Female	Normal hearing	Home	Negative
8	Female	Normal hearing	In-patient	Negative
9	Male	Unknown	Home	Negative
10	Male	Mild–moderate SNHL	Out-patient	Negative
11	Female	Profound SNHL	Home	True positive
12	Female	Unknown	Home	Negative
13	Female	Normal hearing	In-patient	Negative
14	Male	Mild CHL	Home	Negative
15	Female	Normal hearing	Home	Negative
16	Female	Moderate SNHL	In-patient	Negative
17	Male	Mild SNHL	Home	False positive
18	Female	Mild SNHL	Home	False positive

Abbreviations: SNHL—sensorineural hearing loss, CHL—conductive hearing loss. In-patient—Auditory Brainstem Response 2 (AABR2) completed before patient before discharge from hospital. Out-patient—AABR2 completed after patient discharge from hospital.

## Data Availability

Additional data are available from the corresponding author upon request.

## Appendix A. Semi-Structured Interview Guideline

**Questions**	**Prompt**
**Opening Questions**	
1. Before I start, can confirm that <<child’s name>> was CMV negative/positive. Is this your understanding? 2. Has <<child’s name>> had their audiology appointment yet? 3. Did you complete the saliva swab at home or in the hospital? 4. Do you have any other children? If yes, how many? 5. Have you heard of CMV before the screening?	
**Experience** 6. How did being offered the additional CMV test make you feel? 7. How did you find the completing CMV test? 8. Did you find anything helpful or unhelpful about the screening? 9. Are you glad that you had <<child’s name>> tested for CMV? Why/why not? 10. (CMV-positive parents) What was your initial experience when you found out about the CMV diagnosis? 11. Is there anything else you think would be helpful for us to know?	How did you find - The way we gave you information about the CMV screening - The process of taking the saliva swab - The process of returning the saliva swab

## Appendix B. Themes and Further Supporting Quotes

**Theme**	**Quotes**
Theme 1: Parents lacked awareness of CMV prior to cCMV screening	*So until we’d done the test and met you, we actually didn’t know what CMV was at all. And I’d obviously never been taught—it had never been talked about with the GP, which, and I think that’s just a consistency with GPs. My friend is pregnant at the moment, and actually as part of the routine bloods, they did the CMV test and explained what CMV was. So, that just didn’t happen with me, so it was a virus that I’d never heard of at the time. (P11)* *No. I heard about CMV when you guys approached, when someone from your team approached us at the hospital. (P3)* *No. I think that was the thing we had found hardest with the screen. I think knowing what CMV was or knowing that the test was important is the main thing. When I was thinking about this after we spoke the other day, and Dad and I were speaking about it, I think it was the-- not knowing what it was, and then obviously the delivery of getting the information, because we didn’t know what CMV was at the time. (P11)* *Before I had been--I had been sent the information in regards to the CMV test. I didn’t know about that. (P18)* *I don’t think it was. Um I just knew that, obviously you have to be careful. Like I did-- mind you with my son, I’d still kiss him. He was toilet trained anyway, but I’d still you know, make sure that hands are washed, and things like that. At work, I would make sure I was wearing gloves when changing nappies. (P16)* *I work in special care. So, I sort of knew about it, when we have babies that we have suspected like IUGR, so we do testing. (P16)* *So I also got a lot of information from my GP, she was really on top of that when I did share care for the pregnancy with giving me CMV information. (P7)* *Yes. So as you know I am a medical researcher, so yes, I am very familiar with it (P7).* *I think it was the-- not knowing what it (CMV) was, and then obviously the delivery of getting the information, because we didn’t know what CMV was at the time (P11).*
Theme 2: Completing the cCMV screening was overall a positive experience	*The manner of the person doing the testing was friendly and aware I think of the challenging nature. I thought the person came across really aware, I suppose and accommodating (P5).* *No, all the experience is actually quite good, surprisingly good. (P11)* *Yeah. Fantastic. It’s not invasive at all. Like, it’s not invasive. You’re just swabbing a cheek. It’s something very simple and quite easy. No poking around in the ear or up the nose doing a nasopharyngeal. You’re literally just scraping the side of the mouth. And it’s with a cotton bud, basically. It’s nothing. It’s quite simple, quite straightforward, easy to do. (P12)* *I do remember being um, like coming away thinking like I remember thinking it was a really good service, which makes me think it would’ve been explained like very, um, like very coherently. So yea, at the time I think it was well explained and was really well approached as well. It is quite a confusing time, postbirth, in those first 48 h. So um yea I remember thinking it was really well managed, well kind of well done. (P5)* *So yeah, I think it’s um, um the nice and easy experience for me, not quite hard to--to understand, so yeah.(P10)* *So um, yeah. I think the girl that was doing it was quite new, um but even still, she explained everything um well. My partner was there too, so he obviously needed a little bit more explanation, I guess. Um but yeah, overall, fine. Yes. The experience was positive. (P16)* *And I don’t think it can be any simpler than what it is. You’ve got a stick in there. You’ve got uh, the process of how to do it, and what you’ve got to do, and step-by-step even to mailing it. And you’ve brought the mail in there as well. Now, you’ve got the stamps. You don’t have to get anything but take it out, swab your child’s mouth, put it in the sterile department, close it, write his name, birthdate, time and date of collection, address, and you’re done. (P12)* *Um, really simple. Really easy in terms of getting the saliva from Baby… It didn’t appear to cause any discomfort. It didn’t cause um, any trauma for him. For him, it was just waiting for a feed and there’s something in my mouth. Getting it back into its container was easy. Not an issue. Um, and then you drop it in the post box. It can’t get much easier than that. Everything was pre-labelled, pre-done for you. That makes it 1000 times easier when you’ve got 100 things on your mind already (P15)* *The test itself is quite straightforward, it’s just a swab. Um it wasn’t intrusive or anything like that, so I’m quite comfortable with doing that test anyway on Baby (P8)* *And they made it sound like it was a process. It wasn’t really anything that would help (baby) in the long run (P11).*
Theme 3: Parents’ understanding of CMV post cCMV screening varied	*Yes I understand that the idea was to find the cause of hearing loss. (P1)* *It’s basically a virus that you get, but you don’t even know you have it, and it can be passed on to the child and cause hearing loss. (P2)* *That yes, in some cases, apparently, it can cause cerebral palsy, and also in some cases, it can be associated with hearing issues, hence the screening test that we did for my second daughter. (P13)* *My understanding is that it is, I thought it might’ve been a herpes virus. (P6)* *So, it’s a virus that can be sort of picked up, mainly I think from like faecal matter in cats and also from children, uh young children. It’s quite rare, I guess, but if you contract it in um pregnancy, it can be quite, uh I’d say dangerous, I guess, dangerous to the unborn foetus. Um, there’s obviously complications like hearing loss, brain damage, um small baby’s liver damage, um and things like that. Um, yeah. It’s only more that if the pregnant mom contracts it while being pregnant, um not necessarily if she’s had it before being pregnant. (P16)* *Not very much. Just that it does impact babies’ hearing. And if it had been tested while I was pregnant it could have been treated um, from the very start and it wouldn’t have been an issue. And it has to be picked up in the first 14 days in order for treatment to be effective. (P15)*
Theme 4: Parents were glad to screen their baby for cCMV	*It was helpful that doing the test rules out one of the causes of hearing loss and about getting more information about why there is a hearing loss. (P1)* *So it has helped me um understand more uh Baby’s condition, and it helped me understand more about how the uh hearing loss come from. I know it doesn’t come from the CMV, but at least that’s one factor uh that I can uh understand better. (P10)* *We feel positive. Like Baby’s in such a good place, and we feel so thankful that he was screened for CMV and that we did get to do the treatment. Because I think otherwise, we probably would’ve lived waiting for worse. (P11)* *Well, if there’s anything wrong with my son, I want to know. And if there’s anything potentially that can happen like-- of course, of course. It’s your child. You want to know if there’s anything happening at all. (P12)* *Yeah. It was good to cross off one potential condition of what might be causing her difficulties. (P13)* *Yes, I am. I’ve really pleased to know that it wasn’t positive in his case and that it’s something that we could rule out to what’s causing his hearing loss or what was causing his hearing loss. (P15)* *I think it’s just more reassurance, I guess, that that wasn’t the cause of um her hearing loss. And I guess it’s one less thing that um … one thing we can tick off and say, right, what else is it? … Um so, yeah. I guess, yeah. Just more reassurance. (P16)* *I’m very impressed, I am very happy it is done and I’m very glad we have identified something to now look at a future directive… (P17)* *Um … Firstly like reassured. Um so there was obviously, Baby had… I think he failed the first two hearing tests. I, so I think it was in a sense, reassuring to also have an extra check for something else done. It was definitely reassuring. Um, I think the other probably emotion would probably be grateful in a sense, because I think I might have said this previously, we were really grateful for the services we had been offered throughout the entire pregnancy, including the CMV screen. (P5)*
	Abbreviations: Cytomegalovirus, CMV. General practitioner, G.
